# Pre-Pectoral Polyurethane Implant Reconstruction Following Batwing Skin-Reducing Mastectomy: A Single-Center Study

**DOI:** 10.3390/jcm15083110

**Published:** 2026-04-19

**Authors:** Alessandra Veronesi, Edoardo Caimi, Gianmaria Ceglia, Federico Giovagnoli, Lavinia Galliera, Nicoletta Denami, Roberta Comunian, Mattia Federico Cavallero, Simone Furlan, Riccardo Di Giuli, Flavio Bucci, Francesco Klinger, Stefano Vaccari, Valeriano Vinci

**Affiliations:** 1 IRCCS Humanitas Research Hospital, Via Manzoni 56, 20089 Rozzano, Milan, Italy; alessandra.veronesi@humanitas.it (A.V.); riccardo.digiuli@humanitas.it (R.D.G.); valeriano.vinci@humanitas.it (V.V.); 2 Department of Medical Biotechnology and Translational Medicine BIOMETRA, Reconstructive and Aesthetic Plastic Surgery School, University of Milan, Via Festa Del Perdono 7, 20122 Milan, Italy; edoardo.caimi@unimi.it (E.C.); gianmaria.ceglia@unimi.it (G.C.); federico.giovagnoli@unimi.it (F.G.); lavinia.galliera@unimi.it (L.G.); nicoletta.denami@unimi.it (N.D.); roberta.comunian@humanitas.it (R.C.); mattia.cavallero@humanitas.it (M.F.C.); simone.furlan@humanitas.it (S.F.); flavio.bucci@unimi.it (F.B.); 3 Department of Biomedical Sciences, Humanitas University, Via Rita Levi Montalcini 4, 20072 Pieve Emanuele, Milan, Italy; 4 Department of Health Sciences, Ospedale San Paolo, University of Milan, Via Antonio di Rudinì, 8, 20142 Milan, Italy; francesco.klinger@unimi.it

**Keywords:** breast cancer, breast reconstruction, implant-based breast reconstruction, skin-reducing mastectomy, batwing mastectomy, direct-to-implant

## Abstract

**Background**: Pre-pectoral direct-to-implant breast reconstruction is increasingly adopted after mastectomy because it avoids pectoralis major dissection, reduces postoperative pain, and eliminates animation deformity. However, reconstruction in patients with large or markedly ptotic breasts remains challenging because of skin envelope management, nipple–areola complex (NAC) viability, and implant stability. This study evaluated batwing skin-reducing mastectomy with immediate pre-pectoral polyurethane-coated implant reconstruction. **Methods**: We conducted a retrospective single-center study of consecutive patients who underwent batwing skin-reducing mastectomy with immediate pre-pectoral polyurethane-coated implant reconstruction between November 2022 and January 2025. Demographic, oncologic, operative, postoperative, and BREAST-Q data were collected. Primary outcomes included complications, oncologic events, and 12-month patient-reported outcomes. **Results**: Thirteen patients underwent reconstruction, accounting for 18 breasts, with a mean follow-up of 12.85 months. Mean age was 54.5 ± 9.7 years, mean body mass index was 27.0 ± 3.4 kg/m^2^, and mean Regnault ptosis grade was 3.46 ± 0.52. No seromas or oncologic recurrences were observed. One hematoma and one late infection requiring implant removal occurred. Superficial NAC/central flap epidermolysis developed in four patients and resolved conservatively; no full-thickness NAC necrosis occurred. BREAST-Q scores improved significantly in all domains at 12 months, including satisfaction with breasts, psychosocial well-being, physical well-being, and sexual well-being (all *p* < 0.05). **Conclusions**: Batwing skin-reducing mastectomy with immediate pre-pectoral polyurethane implant reconstruction appears safe and reproducible in selected patients with advanced ptosis, with acceptable complication rates and significant improvement in patient-reported outcomes.

## 1. Introduction

Breast cancer remains the most frequently diagnosed malignancy among women worldwide, and mastectomy continues to represent a cornerstone of both therapeutic and risk-reducing treatment strategies. Immediate breast reconstruction and direct-to-implant (DTI) has become an integral component of modern breast cancer care, providing well-established benefits in terms of psychological well-being, body image, and quality of life [1,2]. In parallel with advances in oncologic safety, reconstructive techniques have progressively evolved toward approaches aimed at minimizing surgical morbidity while preserving native anatomy.

In recent years, pre-pectoral implant-based DTI breast reconstruction has gained increasing acceptance as a valid alternative to the traditional subpectoral approach [3,4]. By avoiding pectoralis major muscle dissection, the pre-pectoral technique reduces postoperative pain, eliminates animation deformity, and allows for a more anatomical restoration of the breast mound [5]. While initially reserved for patients with small, non-ptotic breasts, growing experience and improved patient selection have expanded the indications for pre-pectoral reconstruction, including selected patients with large and severe breast ptosis. This condition is more common in patients with higher BMI values, which acts as a risk factor for reconstruction failure [6]. Nevertheless, pre-pectoral reconstruction in ptotic breasts remains technically demanding, primarily due to challenges in skin envelope management and implant stabilization [7].

Skin-reducing mastectomy techniques have been developed to address excess skin and reshape the breast envelope in patients with large or ptotic breasts. Although several patterns have been described, these approaches are often associated with specific limitations, including increased tension on mastectomy flaps, compromised vascularity of the nipple–areola complex, and a higher risk of wound-healing complications. Moreover, many skin-reducing techniques were originally conceived for subpectoral reconstruction and may not optimally address the unique requirements of implant stability and soft-tissue support inherent to the pre-pectoral plane [8,9].

Within the reconstructive setting, the choice of implant surface is of paramount importance. Polyurethane-coated implants offer distinct biomechanical properties, such as enhanced tissue adherence and reduced implant mobility, which may provide improved positional stability in the absence of muscular coverage, both for reconstructive and esthetic breast surgery [10,11]. These features render polyurethane implants particularly attractive for pre-pectoral reconstruction, especially in skin-reducing procedures where control of the implant–skin interface is critical [12]. However, clinical evidence supporting their use in this specific context remains limited.

The batwing skin-reducing pattern represents a valuable option in selected patients with large ptotic breasts, allowing for effective reduction in the skin envelope while preserving central breast vascularity and, when oncologically appropriate, enabling nipple-sparing mastectomy [13]. Wise pattern reduction is also a valuable option with reported failure rates of about 12% [14]. Despite its potential advantages, the literature lacks focused analyses on the combination of the batwing pattern with pre-pectoral placement of polyurethane-coated implants. Data addressing surgical safety, esthetic outcomes, and patient-reported satisfaction in this specific reconstructive scenario are currently sparse.

While the batwing skin-reducing technique is well described in oncoplastic breast surgery, its application in combination with immediate pre-pectoral reconstruction using polyurethane-coated implants in patients with advanced breast ptosis has not been extensively investigated. This study aims to describe the surgical technique and to evaluate the feasibility, early clinical outcomes, and patient-reported outcomes of batwing skin-reducing mastectomy followed by immediate pre-pectoral reconstruction using polyurethane-coated implants in a selected cohort of patients.

## 2. Materials and Methods

### 2.1. Study Methods

This retrospective study evaluated patients who underwent skin-reducing mastectomy using a batwing pattern with immediate pre-pectoral breast reconstruction using polyurethane-coated implants at Humanitas Research Hospital between November 2022 and January 2025. All procedures were performed by the same surgical team using a standardized oncologic and reconstructive protocol. The study was conducted in accordance with the Declaration of Helsinki and approved by the local Institutional Review Board/Ethics Committee [15]. All patients provided informed consent for surgery and for the use of anonymized clinical data and photographic documentation for research and publication purposes. Analyses were conducted at both patient and breast level.

### 2.2. Patient Selection

Patients were considered eligible if they underwent therapeutic or risk-reducing mastectomy with immediate implant-based reconstruction using a batwing skin-reducing pattern and pre-pectoral placement of a polyurethane-coated implant. This reconstructive approach was indicated in patients presenting with breast ptosis and/or redundant skin envelope requiring skin reduction, in whom implant-based pre-pectoral reconstruction was considered appropriate after multidisciplinary evaluation. Advanced breast ptosis was defined according to the Regnault classification [16] as grade III or higher, corresponding to a nipple–areola complex positioned significantly below the inframammary fold and at or below the lower contour of the breast. This approach was preferentially selected in patients in whom preservation of the inframammary fold and avoidance of a T-junction were considered advantageous. The batwing pattern was particularly indicated in cases where vertical skin excess was more pronounced than circumferential redundancy, allowing for targeted skin reduction with controlled nipple repositioning.

Relative contraindications included poor skin quality, compromised vascularity, active smoking, significant comorbidities affecting wound healing, and oncologic conditions precluding nipple preservation or requiring more extensive skin excision. In patients with marked circumferential skin redundancy or requiring more aggressive reshaping of the breast base, alternative techniques such as Wise pattern mastectomy were considered more appropriate. Patients undergoing delayed reconstruction, subpectoral implant placement, reconstruction with non-polyurethane-coated implants, or autologous flap-based reconstruction were excluded. Cases with incomplete clinical records or follow-up shorter than 12 months were also excluded from analysis.

### 2.3. Data Extraction and Outcomes

Preoperative assessment included standardized oncologic and reconstructive evaluation. Recorded baseline variables included age, body mass index, smoking status, diabetes, receipt of neoadjuvant therapy, tumor histology, stage, grade, and tumor location. Adjuvant chemotherapy and radiotherapy were also documented, as well as the performance of contralateral prophylactic surgery when applicable. Preoperative breast morphology was assessed clinically, and breast ptosis was graded according to the Regnault classification [16] and recorded in the study database.

Operative variables extracted from surgical reports and the institutional database included type of mastectomy, being either therapeutic or prophylactic, reconstructive procedure, implant characteristics, implant plane, implant volume, reconstructive operative time, drain placement and duration, and length of hospital stay.

Postoperative follow-up was performed at regular intervals according to institutional protocol at 3, 6 and 12 months, and total follow-up duration was recorded. Postoperative complications included seroma, hematoma, surgical site infection, late infection (defined as an infection occurring more than 30 days after surgery and requiring implant removal) and skin flap ischemia or cutaneous suffering. Oncologic outcomes included documented local or regional recurrence during follow-up.

Patient-reported health-related quality of life outcomes were assessed using the BREAST-Q Reconstruction Module [17]. Baseline and twelve-month postoperative BREAST-Q scores were collected and recorded in the database.

### 2.4. Surgical Technique

All procedures were performed jointly by the oncologic and reconstructive surgical teams. Under general anesthesia, patients were positioned supine with both arms abducted at approximately 90°.

**Figure 1:**
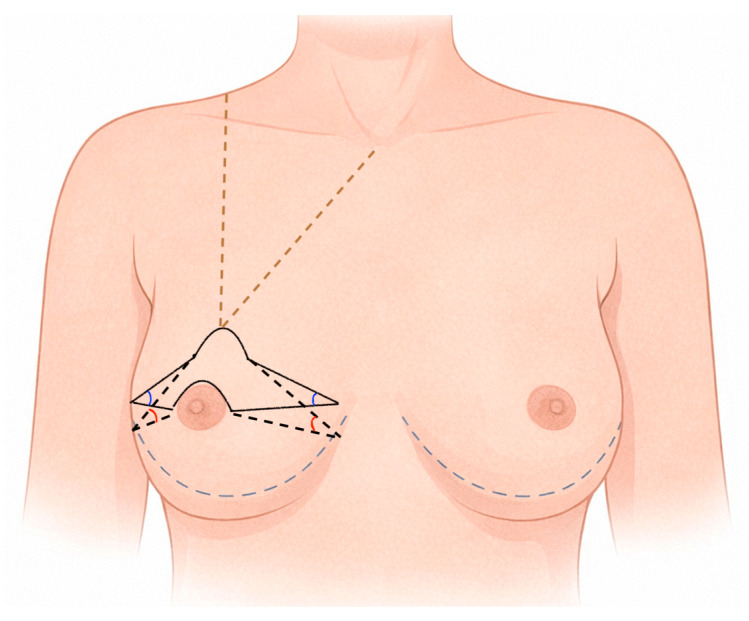
Preoperative design of batwing skin-reducing mastectomy with nipple–areola complex (NAC) repositioning. Brown dotted lines indicate the midclavicular line and the sternum–nipple line, used as anatomical references for breast meridian and NAC positioning. Black solid lines represent the planned incision pattern of the batwing design. The degree of nipple ascension can be modulated by adjusting the angle of the lateral incision limbs: a wider angle (red) results in greater nipple elevation.

Preoperative marking (Figure 1) is a critical step in the batwing skin-reducing technique. The design is performed with the patient in the standing position. The hemisternum, anterior axillary line, inframammary folds, and the arcs of the upper quadrants corresponding to the natural termination of the breast are marked. These landmarks help define the starting point of the “beak” of the mastectomy at the upper poles, providing guidance for the breast surgeon and minimizing the risk of step-off deformity at the junction between resected and preserved tissues.

Planning begins with the positioning of the nipple–areola complex (NAC). The ideal NAC location is generally estimated at 21–23 cm from the sternal notch or approximately at the midpoint of the humeral length. However, given the relative fragility of the skin flaps and their vascular supply, mainly dependent on the dermal plexus and perforating branches of the intercostal vessels, a conservative approach is preferred. Excessive cranial repositioning may increase closure tension and compromise flap viability. Therefore, post-mastectomy skin retraction was considered, and NAC positioning is individualized to minimize traction on the suture line while preserving acceptable esthetic proportions.The definitive NAC position is determined after selection of the implant, particularly in relation to its height. An initially conservative design in terms of NAC elevation allows for intraoperative adjustment based on implant choice and the final positioning of the new inframammary fold.

The length of the inferior pole is assessed preoperatively using a pinching test and directly influences the length of the batwing arms. The new inframammary fold is planned accordingly, with the aim of shortening the inferior pole to an ideal length of approximately 6–7 cm, depending on breast size and implant volume. An excessively long inferior pole may lead to downward tilting of both the implant and the NAC, with the nipple potentially falling below the bra cup. In such cases, repositioning of the inframammary fold is often required and can be achieved through dermal-to-costal periosteum sutures.

The arms of the batwing design should ideally extend to the anterior axillary pillar. Their terminal shape may be tapered or arrow-shaped, depending on the desired degree of breast base reduction. Proper calibration of the distance between the arms allows for adequate skin reduction while preserving soft-tissue vascularity and avoiding excessive tension, which is particularly relevant in the reconstructive setting.

The width of the batwing pattern, defined by the distance between its arms, can be adjusted to achieve modest modification of the breast base. This adjustment depends on the inclination of the lateral arm: a more oblique lateral extension allows for subtle reshaping of the breast footprint without extensive undermining. This flexibility makes the batwing technique adaptable to different breast morphologies while maintaining predictable closure patterns.

The implant pocket can be tailored by reducing its width according to the selected implant, using sutures between the subcutaneous tissue of the flap and the costal periosteum or muscular fascia. This maneuver allows for reduction and symmetrization of the breast base, contributing to improved implant stability and overall breast shape.

The batwing area may be managed either by full-thickness skin resection or by simple de-epithelialization. Both approaches are described in the literature, and the superiority of preserving a dermal flap remains controversial. De-epithelialization may provide additional dermal support to the reconstruction, whereas complete excision simplifies the procedure and reduces tissue bulk. The choice between these options depends on surgeon preference, reconstructive objectives, and patient-specific factors.

Skin-sparing or nipple-sparing mastectomy was subsequently performed by the breast surgical team through the previously marked batwing incision. During the mastectomy, careful dissection was carried out at the level of the superficial breast fascia in order to preserve the subcutaneous layer and maintain adequate mastectomy flap thickness. Attention was given to preserving dermal vascularity and minimizing trauma to the mastectomy flaps. Flap perfusion was assessed intraoperatively through standardized clinical evaluation, including skin color, capillary refill, dermal bleeding, and tissue turgor. Attention was given to maintaining adequate mastectomy flap thickness through dissection at the level of the superficial fascia, aiming to preserve a minimum thickness of approximately 1 cm. When adequate flap thickness could not be preserved, indocyanine green angiography was utilized. These clinical parameters were used consistently throughout the series to guide intraoperative decision-making regarding flap viability.

Following completion of the mastectomy, the reconstructive phase was initiated. A pre-pectoral pocket was created by gentle release of residual glandular adhesions while preserving the pectoralis major fascia. Implant selection was guided primarily by breast base width and thoracic dimensions, ensuring congruence between the implant footprint and the native breast base. Polyurethane-coated implants were then positioned in the pre-pectoral plane. Implant sizers with breast corresponding width were used to ensure correct placement and breast shape before the definitive implants were positioned.

**Figure 2:**
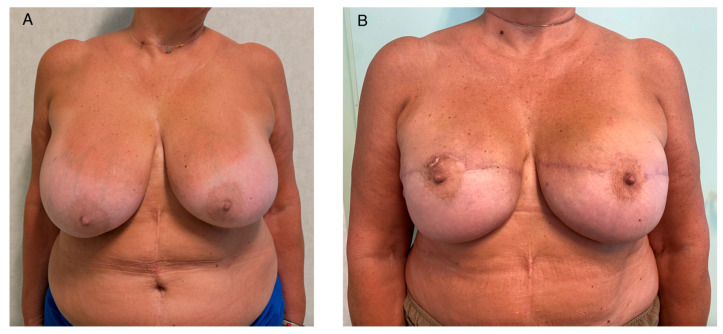
(**A**) Preoperative frontal view showing large asymmetric breasts with a broad base, marked and asymmetric breast ptosis (Grade III), and asymmetric nipple–areola complex (NAC) position. (**B**) Frontal view at 12-month follow-up demonstrating correction of ptosis, improved breast symmetry, improvement in upper pole fullness, satisfactory repositioning of the NACs, and stable implant positioning with appropriate contour and projection.

The reconstructed breast mound was evaluated with the patient temporarily placed in a semi-upright position to assess implant position and pocket stability. When necessary, the implant pocket was tailored with selective interrupted sutures between the flap and the chest wall to optimize implant positioning and prevent lateral displacement, while avoiding excessive tension on the mastectomy flaps (Figure 2).

Definitive skin tailoring was performed only after implant placement and stabilization of the reconstructed breast mound. The excess skin within the batwing design was then excised, allowing for controlled elevation and repositioning of the NAC. This delayed adjustment enabled fine tuning of nipple position and skin envelope tension while preserving flap perfusion.

The incision was closed in layered fashion with absorbable sutures. Closed-suction drains were placed in all cases. Drains were removed when output was less than 30 mL over a 24 h period for three consecutive days, according to institutional protocol.

Contralateral symmetrization procedures were not performed at the time of reconstruction. Surgical planning prioritized oncologic safety and immediate reconstruction, while contralateral procedures were deferred in selected cases to allow for completion of adjuvant treatments and more accurate postoperative assessment of breast symmetry. We believe this staged approach may facilitate more precise correction when needed and reflects common clinical practice in complex reconstructive scenarios.

### 2.5. Statistical Analysis

Statistical analysis was conducted using Microsoft Excel (Microsoft Corporation, Redmond, WA, USA). Continuous variables were described as mean ± standard deviation (SD) and range. The Shapiro–Wilk test was used to verify for normal distribution of continuous variables. Consequently, BREAST-Q scores were analyzed as continuous variables using Student’s *t* test. *p* values less than 0.05 were considered statistically significant.

## 3. Results

### 3.1. Patient Demographics

A total of 13 patients underwent batwing skin-reducing mastectomy followed by immediate pre-pectoral implant-based breast reconstruction. A total of 8 patient underwent unilateral mastectomy, while 5 patients underwent bilateral, risk-reducing mastectomy, accounting for a total of 18 reconstructed breasts. Mean follow-up duration was 12.85 months.

Mean patient age at surgery was 54.5 ± 9.7 years, and mean body mass index was 27.0 ± 3.4 kg/m^2^. Mean ptosis grade was 3.46 ± 0.52. Specific patient demographics are reported in Table 1.

### 3.2. Oncologic Characteristics

Regarding oncologic characteristics, most cases were invasive carcinoma of no special type with luminal subtype predominance, alongside a smaller proportion of triple-negative tumors and ductal carcinoma in situ. Two procedures were prophylactic in BRCA2-positive patients. Oncologic characteristics are reported in Table 2.

### 3.3. Surgical Characteristics and Outcomes

All reconstructions were performed in the pre-pectoral plane using polyurethane-coated implants. Mean implant volume was 474 ± 84 cc with a mean operative time for the reconstructive phase of 98.5 ± 24.1 min. Operative characteristics are illustrated in Table 3.

Complications were classified as major (requiring surgical revision or implant removal) or minor (managed conservatively). No cases of postoperative seroma were observed. One patient (7.7%) developed a hematoma, which was managed by evacuation in an outpatient setting without implant loss. One patient (7.7%) experienced a late surgical site infection that ultimately required implant explantation at 12 months postoperatively.

**Figure 3:**
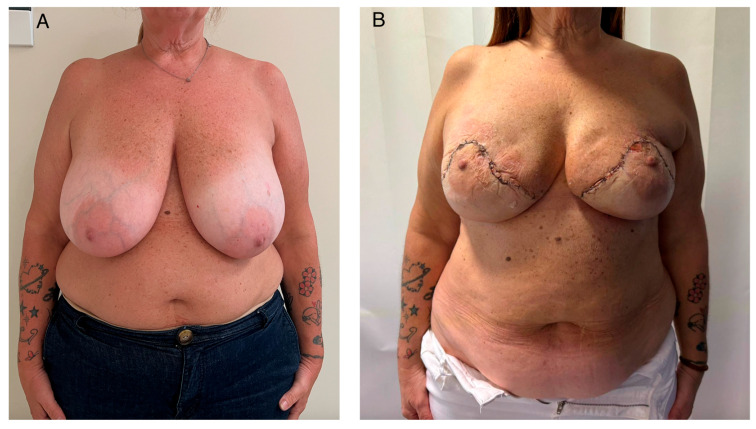
(**A**) Preoperative frontal view showing large breasts with a wide base, empty upper pole and significant breast ptosis (grade III). (**B**) Postoperative view at 1-month follow-up demonstrating correction of ptosis with effective elevation and repositioning of the nipple–areola complex (NAC), resulting in improved breast projection and contour. In this case, partial superficial suffering of the superior aspect of the NAC was observed and managed conservatively, with complete resolution and no further complications.

Events involving the nipple–areola complex and central skin envelope were standardized and classified as superficial NAC/central flap epidermolysis (partial-thickness ischemic suffering). These events were recorded in 4 patients (30.8%) and were managed conservatively with local wound care, with complete secondary re-epithelialization and no need for surgical revision (Figure 3). No full-thickness NAC necrosis or complete nipple loss occurred.

No locoregional or distant oncologic recurrences were observed during the available short-term follow-up period. All complications are reported in Table 4.

### 3.4. Patient-Reported Outcomes

Patient-reported outcomes assessed using the BREAST-Q Reconstruction Module demonstrated significant postoperative improvements across all evaluated domains at 1-year follow-up. Mean satisfaction with breasts increased from 58.4 ± 16.2 to 84.6 ± 6.3, corresponding to a mean increase of 26.2 points. Psychosocial well-being improved by 25.1 points, physical well-being of the chest increased by 17.2 points, and sexual well-being improved by 28.2 points. All changes were statistically significant (*p* < 0.05) (Table 5).

## 4. Discussion

The extension of pre-pectoral implant-based breast reconstruction to patients with advanced breast ptosis remains one of the most debated areas in contemporary implant reconstruction [12,18]. Although the pre-pectoral approach offers recognized advantages, including reduced postoperative pain, preservation of pectoralis major function, and elimination of animation deformity, its application in markedly ptotic breasts is technically demanding [12,19,20]. In this setting, reconstructive success depends not only on oncologic safety, but also on reliable preservation of mastectomy flap perfusion, adequate control of the skin envelope, and stable implant positioning in the absence of muscular coverage or biologic matrices [12,18,19].

Polyurethane-coated implants have regained interest in the pre-pectoral setting because of their tissue-adherent properties and their potential to improve implant stability [21,22]. Polyurethane implants can be used safely in direct-to-implant pre-pectoral reconstruction, including in moderate-to-large breasts, with acceptable complication rates and satisfactory esthetic outcomes [10]. However, most published studies have focused either on standard direct-to-implant cases or on Wise pattern skin-reducing mastectomies [9,12,19,23,24]. By contrast, data specifically addressing the batwing pattern in combination with polyurethane-coated implants in the pre-pectoral plane remain extremely limited. In this regard, the present study addresses a relatively unexplored but clinically relevant reconstructive scenario.

In our series of 13 patients, corresponding to 18 reconstructed breasts, batwing skin-reducing mastectomy followed by immediate polyurethane-based pre-pectoral reconstruction yielded favorable early outcomes despite the inclusion of a reconstructively challenging population characterized by advanced ptosis. No seromas were observed, no cases of full-thickness nipple–areola complex necrosis occurred, and implant loss was limited to one case related to late infection. Patient-reported outcomes were also encouraging. At 1-year follow-up, BREAST-Q scores demonstrated significant improvements across all evaluated domains, including satisfaction with breasts, psychosocial well-being, physical well-being of the chest, and sexual well-being. The mean postoperative satisfaction with breasts reached 84.6, reflecting a substantial improvement compared with baseline values. These findings suggest that effective skin-envelope management combined with stable implant positioning in the pre-pectoral plane can translate not only into acceptable surgical safety but also into meaningful improvements in patient-perceived quality of life.

One of the most relevant observations of this series concerns the management of nipple–areola complex and central flap viability. In skin-reducing mastectomy, particularly in the setting of advanced ptosis, vascular compromise of the central skin envelope remains a major concern [25]. In our cohort, superficial NAC or central flap epidermolysis was observed in four patients, but all cases healed with conservative outpatient treatment and no patient developed complete NAC loss or required revisional surgery for ischemic complications. Importantly the batwing technique allows the surgeon to perform a mastectomy with the ability to preserve the 5th intercostal perforator, which is fundamental for NAC vascularity [26]. These findings are noteworthy as they suggest that the batwing design may permit effective reduction in the skin envelope while preserving adequate central perfusion, provided that flap handling is cautious and perfusion is assessed rigorously throughout the procedure. In the literature, reported rates of NAC necrosis generally range between 2% and 10%; however, these figures often refer to full-thickness necrosis requiring surgical management [12,19,22,23,25]. In contrast, all cases observed in our cohort were limited to partial-thickness epidermolysis, resolved with conservative management, and did not progress to full-thickness necrosis or require surgical revision. No cases of complete NAC loss were observed. These events should therefore be considered minor and transient complications rather than true necrotic outcomes.

The present study also highlights the importance of implant stability and inferior pole control in pre-pectoral reconstruction without ADM. In the absence of muscular coverage, implant position depends entirely on pocket design, the relationship between the implant footprint and the native breast base, and the interaction between the implant surface and the surrounding tissues [27]. Polyurethane-coated implants may be particularly advantageous in this context because their adherent surface may reduce early mobility and contribute to more predictable positional stability [10]. In our experience, the reconstructive result did not rely on aggressive skin tightening alone. Instead, inferior pole refinement was obtained through selective pocket modulation and, when required, selective interrupted sutures between the flap and the chest wall. This strategy reduces dependence on excessive cutaneous tension and may therefore lessen ischemic stress on the mastectomy flaps. The absence of seroma in our cohort may further support the stability of this reconstructive construct.

A further point emerging from our experience is the central role of breast base width in procedural planning. In skin-reducing pre-pectoral reconstruction, implant selection should not be driven by breast volume alone, but rather by congruence between implant footprint and thoracic dimensions. When the implant base width is well matched to the native breast base, vertical skin reduction through a batwing design may be sufficient to restore contour without the need for more extensive transverse excision. Conversely, when a substantial mismatch exists between the desired implant footprint and the native breast envelope, alternative skin-reducing designs may provide better control. In this sense, breast base width should be regarded not only as a tool for implant sizing, but also as a determinant of incision strategy.

Another technically relevant aspect of our approach is the decision to preserve the skin envelope until completion of implant placement and pocket stabilization, with definitive skin tailoring and final NAC repositioning performed only after the reconstructed breast mound has been established. In patients with advanced ptosis, premature skin excision may increase the risk of ischemia or lead to suboptimal nipple position [28]. The absence of full-thickness NAC necrosis in our cohort, despite the degree of ptosis and the need for skin reduction, may in part reflect the value of this conservative intraoperative strategy.

Compared with other skin-reducing techniques such as the Wise pattern, the batwing design offers specific technical advantages in selected patients. In particular, it avoids the creation of a T-junction, which is known to represent a critical point for wound healing complications, and allows for targeted vertical skin reduction while preserving the inframammary fold. These features may be especially advantageous in the pre-pectoral setting, where minimizing flap tension and preserving vascularity are essential for reconstructive success. Furthermore, procedures involving skin-reducing mastectomy combined with immediate implant-based reconstruction are inherently more complex than standard mastectomy and may require longer operative times. In our series, the mean operative time for the reconstructive phase was approximately 98 min, reflecting the additional steps required for implant selection, pocket preparation, and controlled skin tailoring. Although direct comparisons with standard mastectomy were not performed, it should be noted that, in experienced breast units with a well-coordinated oncologic and reconstructive team, operative times may become comparable to those of standard mastectomy due to optimization of surgical workflow and team expertise.

Overall, these technical considerations suggest that the batwing approach should not be viewed merely as an alternative skin pattern, but rather as part of a morphology-based reconstructive strategy. In our view, the suitability of batwing reduction depends on a combination of oncologic feasibility, central skin safety, flap quality, systemic risk profile, base-width compatibility, and the distribution of ptosis. Patients in whom vertical excess and NAC malposition predominate over circumferential redundancy appear particularly well suited to this design, as batwing reduction allows for focused correction while preserving the inframammary fold and avoiding a T-junction. This may be especially advantageous in the prepectoral setting, where minimizing wound tension is essential. In this context, the combination of mastectomy-induced reduction in the skin envelope and the adhesive “Velcro effect” of polyurethane may promote moderate skin envelope contraction, thereby facilitating implant stabilization and favorable soft-tissue redraping [11]. However, these findings should be interpreted in the context of a relatively short follow-up, as longer-term outcomes, including capsular contracture and implant stability, require extended observation beyond the first postoperative year. Furthermore, despite their potential advantages, polyurethane-coated implants also present specific limitations that should be carefully considered. Their strong tissue adherence, while beneficial for implant stability, may render explantation more technically demanding, particularly in the setting of infection, malposition, or revision surgery [19,23,24]. This characteristic may increase operative complexity and requires appropriate surgical experience [24]. In addition, polyurethane-coated implants are generally associated with higher costs compared with standard textured or smooth implants, which may limit their widespread adoption in certain healthcare settings [12,19,23,24]. Furthermore, although available evidence suggests favorable outcomes in both esthetic and reconstructive breast surgery, long-term data specifically addressing their performance in the pre-pectoral setting, particularly in patients with large or ptotic breasts, remain relatively limited [12,19]. As such, caution is warranted when extrapolating early results to long-term outcomes, and further studies with extended follow-up are needed to fully define their role within contemporary implant-based breast reconstruction.

### 4.1. Limitations

Our findings should be interpreted in light of different limitations. The retrospective design inherently introduces the risk of selection bias, and the sample size remains small. The small sample size reflects the selective nature of the indication and limits the statistical power and generalizability of the findings. The mean follow-up of 12.85 months allows for the assessment of early clinical and patient-reported outcomes; however, it remains insufficient to draw definitive conclusions regarding long-term endpoints such as capsular contracture, late implant malposition, and the durability of esthetic results. In implant-based breast reconstruction, these complications may manifest beyond the first postoperative year, and a minimum follow-up of at least 24 months is generally recommended to provide a more comprehensive evaluation of long-term reconstructive stability. In addition, the absence of a comparative cohort prevents direct evaluation of the batwing pattern against alternative skin-reducing approaches, particularly Wise pattern mastectomy. The absence of a comparative cohort represents an inherent limitation of this study and precludes direct assessment of the relative performance of the batwing approach compared with alternative techniques, including Wise pattern mastectomy, ADM-assisted reconstruction, or subpectoral implant placement. However, when interpreted in the context of the existing literature, the complication profile observed in our series appears broadly consistent with previously reported outcomes in patients with large or ptotic breasts undergoing implant-based reconstruction. Rates of minor ischemic complications and overall implant loss remain within the range described in comparable reconstructive settings. Nevertheless, the homogeneity of the reconstructive technique, the exclusive use of polyurethane-coated implants in the pre-pectoral plane, and the focus on a clearly defined subgroup of patients with advanced ptosis strengthen the internal consistency of the series.

### 4.2. Future Perspectives

Future investigations should aim to further define the role of the batwing skin-reducing pattern within the expanding spectrum of pre-pectoral implant-based reconstruction. Prospective studies with larger patient cohorts and longer follow-up will be necessary to evaluate long-term reconstructive stability, capsular contracture rates, and the durability of esthetic outcomes in this specific population of patients with advanced breast ptosis. Comparative analyses between the batwing approach and other skin-reducing techniques, particularly Wise pattern mastectomy, may help clarify the relative advantages of each design in terms of flap perfusion, complication profiles, and esthetic control of the breast envelope. In addition, future research integrating objective esthetic assessment, three-dimensional surface analysis, and longitudinal patient-reported outcomes could provide a more comprehensive evaluation of reconstructive success. As indications for pre-pectoral reconstruction continue to expand, further refinement of patient selection criteria and surgical planning algorithms will likely play a key role in optimizing outcomes in patients with large or ptotic breasts.

## 5. Conclusions

This study provides preliminary evidence that batwing skin-reducing mastectomy combined with immediate pre-pectoral reconstruction using polyurethane-coated implants appears to be a feasible and safe approach in the short term, although these findings should be considered preliminary and require confirmation in larger studies. The technique appears to offer satisfactory skin envelope control, preservation of NAC viability, and stable implant-based reconstruction without the routine use of ADM. Larger comparative studies with longer follow-up will be necessary to clarify the role of this approach within the broader spectrum of skin-reducing pre-pectoral reconstruction.

## Figures and Tables

**Table 1:** Patient sample demographics.

Variable	Value
Patients, n	13
Reconstructed breasts, n	18
Age, mean ± SD (range), years	54.5 ± 9.7 (39–70)
BMI, mean ± SD (range), kg/m^2^	27.0 ± 3.4 (20.69–32.05)
Active smokers, n (%)	2 (15.4%)
Diabetes mellitus, n (%)	1 (7.7%)
Regnault ptosis grade, mean ± SD (range)	3.46 ± 0.52 (3–4)
Contralateral prophylactic mastectomy, n (%)	5 (38.5%)

**Table 2:** Oncologic characteristics of included patients.

Case	Cancer Definitive Histology	TNM Stage	Grade	Neoadjuvant Chemotherapy	Adjuvant Therapy (Which Type)	Adjuvant Radiotherapy
1	Luminal infiltrative carcinoma, non special histotype. Luminal B	T1c N1a M0	G2	Yes	Yes (Letrozole + Abemaciclib)	Yes
2	Luminal infiltrative carcinoma, non special histotype. Luminal B	T1c N1a M0	G3	No	Yes (Letrozole + Abemaciclib)	No
3	Luminal infiltrative carcinoma, non special histotype. Luminal A	T1b N1a M0	G2	No	Yes (Letrozole + Abemaciclib)	No
4	Luminal infiltrative carcinoma, non special histotype. Luminal A	T2 N0 M0	G2	No	Yes (Letrozole + Abemaciclib)	Yes
5	Luminal infiltrative carcinoma, non special histotype. Triple Negative	T1 N0 M0	G3	Yes	No	No
6	Ductal in situ carcinoma.	Tis N0 M0	G3	No	No	No
7	Luminal infiltrative carcinoma, non special histotype. Luminal B	T1b N1a M0	G3	Yes	No	No
8	Luminal infiltrative carcinoma, solid–papillary histotype. Luminal B	T1a N0 M0	G2	No	No	No
9	Luminal infiltrative carcinoma, non-special histotype. Luminal B	T1c N0 M0	G3	No	Yes (Letrozole)	No
10	Luminal infiltrative carcinoma, non-special histotype. Triple Negative	T1 N0 M0	G3	Yes	No	No
11	Luminal infiltrative carcinoma, non-special histotype. Luminal A	T1 N0 M0	G2	No	No	No
12	Prophylactic- BRCA2+	NA	NA	No	No	No
13	Prophylactic- BRCA2+	NA	NA	No	No	No

**Table 3:** Operative Characteristics of Included Patients.

Variable	Value
Implant plane	Pre-pectoral (100%)
Implant type	Polyurethane-coated (100%)
Implant volume, mean ± SD (range), cc	474 ± 84 (220–560)
Operative time, mean ± SD (range), min	98.5 ± 24.1 (56–150)
Length of stay, mean ± SD (range), days	2.23 ± 0.83 (1–3)
Drain removal time, mean ± SD (range), days	16.6 ± 4.4 (14–27)

**Table 4:** Complications of included patients.

Variable	Value
Seroma, n (%)	0 (0%)
Hematoma, n (%)	1 (7.7%)
Surgical site infection, n (%)	1 (7.7%)
Implant loss, n (%)	1 (7.7%)
Superficial NAC/central flap epidermolysis, n (%)	4 (30.8%)
Full-thickness NAC necrosis, n (%)	0 (0)
Oncologic recurrence, n (%)	0 (0)

**Table 5:** Results of Baseline and 1-Year Postoperative BREAST-Q Evaluation.

Patient Reported Outcomes Measures	Baseline		1 Year Postoperative		Mean Difference	*p* Value
	Number of Patients	Baseline Mean ± SD	1-Year Postoperative	Number of Patients		
Satisfaction with breasts	13	58.4 ± 16.2	13	84.6 ± 6.3	+26.2	<0.05
Psychosocial well-being	13	61.2 ± 15.8	13	86.3 ± 9.4	+25.1	<0.05
Physical well-being (chest)	13	63.5 ± 14.1	13	80.7 ± 11.2	+17.2	<0.05
Sexual well-being	13	50.7 ± 17.5	13	78.9 ± 12.6	+28.2	<0.05

## Data Availability

Data availability is not publicly available but can be requested to the corresponding author.

## Abbreviations

The following abbreviations are used in this manuscript:

NAC	Nipple-Areola Complex
DTI	Direct-To-Implant
SD	Standard Deviation
